# Invisible Emergency: Stress and Anxiety-Depressive Disorders among Medical Students in Tunisia

**DOI:** 10.1192/j.eurpsy.2026.11422

**Published:** 2026-07-31

**Authors:** R. Kammoun, H. Hsine, H. Ghabi, M. Mehdi Karoui, O. Mechergui, F. Ellouze

**Affiliations:** 1 Psychiatry G, Razi Hospital, Tunis, Tunisia; 2 Psychiatry, La Metairie Clinic, Nyon, Switzerland

## Abstract

**Introduction:**

Medical training exposes students to high levels of stress, associated with significant risks of depression and anxiety. While this impact has been extensively studied worldwide, it remains underexplored in Tunisia.

**Objectives:**

To estimate the prevalence of psychological disorders among medical students enrolled in the four Tunisian faculties of medicine.

**Methods:**

A descriptive cross-sectional study was conducted online using a self-administered Google Forms questionnaire over a two-month period (October–November 2024). Stress was assessed using the PSS-10 scale, anxiety and depressive symptoms with the HADS scale, and sleep quality with the PSQI score.

**Results:**

We included 643 participants. The mean PSS score was 33.16 ± 6.58, with 80.6% exhibiting a high level of perceived stress. Mean HADS-A and HADS-D scores were 10.46 ± 4.22 and 8.37 ± 3.96, respectively. Anxiety-depressive disorders were reported in 25.3% of participants. Poor sleep quality was observed in 69.1% of students, with a mean PSQI score of 7.46 ± 3.3.

**Conclusions:**

Stress and anxiety-depressive symptoms are highly prevalent among Tunisian medical students. Preventive strategies should be implemented at the faculty and ministerial levels.

**Disclosure of Interest:**

None Declared

